# Improving viability of leukemia cells by tailoring shell fluid rheology in constricted microcapillary

**DOI:** 10.1038/s41598-020-67739-3

**Published:** 2020-07-14

**Authors:** Mohammad Nooranidoost, Ranganathan Kumar

**Affiliations:** Department of Mechanical and Aerospace Engineering, University of Central Florida, Orlando, FL USA

**Keywords:** Cell biology, Engineering, Physics

## Abstract

Encapsulated cell therapy has shown great potential in the treatment of several forms of cancer. Microencapsulation of these cancer cells can protect the core from the harmful effects of the neighboring cellular environment and can supply nutrients and oxygen. Such an encapsulation technique ensures cell viability and enables targeted drug delivery in cancer therapy. The cells immobilized with a biocompatible shell material can be isolated from the ambient and can move in constricted microcapillary. However, transportation of these cells through the narrow microcapillary may squeeze and mechanically damage the cells which threaten the cell viability. The cell type, conditions and the viscoelastic properties of the shell can dictate cell viability. A front-tracking numerical simulation shows that the engineered shell material with higher viscoelasticity improves the cell viability. It is also shown that low cortical tension of cells can contribute to lower cell viability.

## Introduction

Cancer cell mechanics have been extensively studied in microfluidic systems for cell sorting^1^, cell banking^2^ and cancer therapy^3^. Several research papers have classified cancer cells based on their mechanical properties, which can be utilized for the development of innovative diagnostic devices^3–6^. The cancer cell deformation has been studied as a tool to sort healthy and unhealthy cells for cancer therapies and to disrupt the metastatic process^3^. Microfluidic optical stretchers can deliver individual cells^7^. The experiments on malignant transformation of human breast epithelial cells characterized the relationship between cellular function and cytoskeletal mechanical properties. The cancer cells may deform up to five times more than the healthy cells and that the metastatic cancer cells have a tendency to deform more than the non-metastatic cancer cells^7^. Encapsulation of various cancer cells has been done over long culture periods using tumor microsphere and spheroids models^2^. This study showed a higher uniformity and lower variability in diameter and circularity of the tumor microspheres over self-aggregated tumor spheroids, however both models can assure high cell viability. A mathematical model also has been developed to characterize the cellular interaction with endothelium that showed a larger deformation of cancer cells compared to healthy cells during metastasis^5^. Thus, while encapsulation techniques in microchannels improve cell viability and target delivery, it is important to study the deformation of encapsulated leukemia cells in constricted microchannels.

Although numerous experiments have been conducted in cell biophysics, modeling simulations of cell mechanics in bio-microfluidic systems have been lacking largely due to the complexity of the system. Lykov et al.^8^ reviewed recent modeling approaches for cell mechanics simulations. It is possible to develop computational models to study the flow phenomena for different suspended cells such as white blood cells (WBC), circulating tumor cells (CTC) and other cancer cells. The effects of channel geometry and cell properties on deformation of CTC passing through a micro-filtering channel show that the channel cross-section as well as cell elasticity characterize the splitting and separation of cells as well as the cell deformation^9,10^. Margination^11,12^ and adhesion^13^ of CTC and WBC have also been studied which shows a firm adhesion of cells in a small capillary. Our team modeled the encapsulation of single cells in flow focusing geometries^14,15^ and deformation of encapsulated cells in bioprinting systems^16^, identified various modes of encapsulation and showed that the microfluidic device^15^ needs to be carefully designed to achieve a high success rate of single cell encapsulation. The fluid properties can also influence encapsulation^14^. Our work on bio-printing systems also examined the importance of bio-ink viscoelasticity on cell deformation and survivability of the cell^16^. It is clear that the cell viability can be improved by adding polymers to the bio-ink fluid.

Droplet-based microfluidics facilitates cell deposition^16,17^, cell encapsulation^14,18^ and cell sorting^19^ in tissue engineering. Controllability characteristics of these systems can be exploited to optimize the process and prevent undesired and harmful phenomena by adjusting physical conditions and relevant parameters. Cell viability can be optimized during deposition by describing the viability as a function of impingement parameters^17^. The limitation of flow cytometry for high-throughput analysis of cell sorting can be overcome by compartmentalization of single cells in droplets^19^, and experiments have shown that cells that are protected by a hydrogel cell carrier are more viable during the syringe needle flow compared to those covered by a Newtonian fluid^20^. A similar finding is observed for viability of single cells during the deposition process in bioprinting systems^16^, where the cells and bio-inks are modeled using a viscoelastic model to capture rheological behavior of the fluids. These cells were encapsulated by polymeric fluids consisting of long-chain molecules in their mixture, in which the polymer concentration can be tailored to reach the desired design^16^. Therefore, the literature makes a strong case for modeling polymeric fluids for migration of encapsulated cells.

Migration of single cells in constricted microchannels has been extensively studied in recent years to examine selective entrapping^21^, metastatic potency^22^ and passage behavior^23^ of biological cells in microchannels with different constrictions. However, the effect of encapsulating single cells with polymeric fluids on cell deformation and its impact on their viability is still unexplored. This study focuses on the deformation of encapsulated leukemia cells migrating in a constricted microchannel. The objective of this work is to delineate the impact of the encapsulating droplet on deformation and the viability of three different types of leukemia cells including HL60, Neutrophil and Jurkat. These cell types are chosen for their variation in size and cortical tension. An in-house code is used to model cells as viscoelastic liquid droplets. A FENE-CR (Finite Extendable Nonlinear Elastic-Chilcott and Rallison) viscoelastic model is accounted for viscoelasticity of the cells. In this model, total viscosity is defined as the sum of the solvent and polymeric viscosities. Cell solvent viscosity is related to the viscosity of cytosol and cell polymeric viscosity to the viscosity of the cytoskeleton. The FENE-CR model is chosen here because it can represent relaxation mechanism and finite extensibility of protein-based components of the cell. Polymers in the FENE-CR model are modeled as finitely extensible mass-spring dumbbells in a solution, so it is deemed to be more realistic than the Maxwell and Oldroyd-B models where the polymers are assumed to be infinitely extensible^41^.

Polymeric concentration of the shell fluid is an important property in characterizing the rheological behavior of the flow and dynamics of cell migration in constricted microcapillary. Altering this fluid property of the shell fluid will manipulate the cell shape deformation, which may lead to cell death. In this work, we tailor the shell fluid polymeric concentration by adjusting the amount of polymers in the shell fluid to reduce the cell mechanical deformation. The cell deformation would then be related to cell viability by using a theoretical cell survival model quantitatively calculate the viability of the cells. Simulations are done for different polymeric viscosity ratio to reach a low mechanical deformation for all three leukemia cells, which assure a high cell viability.

## Results

**Figure 1:**
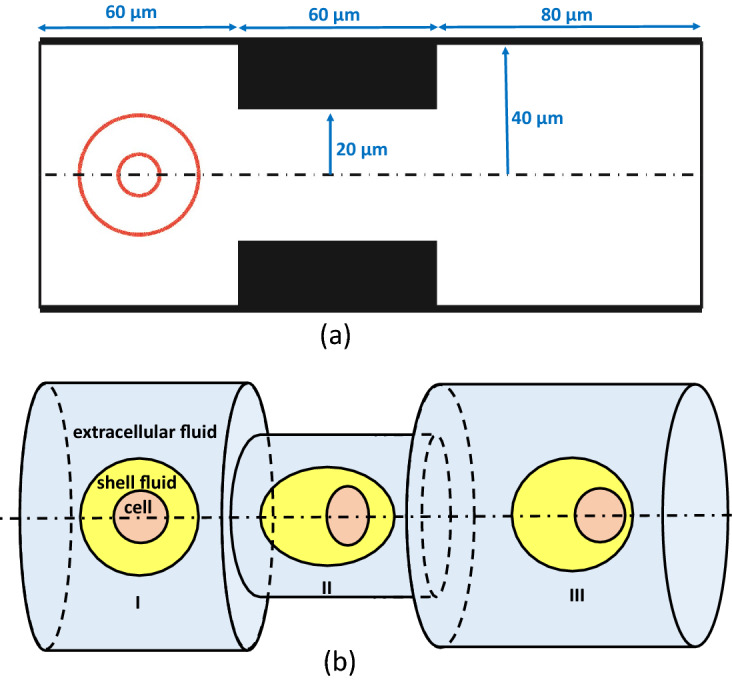
(**a**) Computational setup. (**b**) Schematic illustration of the simulation setup. In region II, both cell and shell fluid droplet deform while passing through the contraction. The cell inside the encapsulating droplet moves faster than the droplet due to the pressure loss and therefore, the cell moves to the front end of the droplet.

Simulation of flow dynamics and motion of leukemia cells transiting through a constricted microcapillary is performed to determine the deformation and viability of leukemia cells under the extensional flow in the microchannel. The microchannel has a circular cross section, with diameter (D = 80 μm), and a cylindrical contraction in the middle of the channel, which narrows the channel cross section (Fig. 1a). The schematic illustration of the cell-laden droplet shape evolution shows three different stages: (I) before contraction, (II) within the contraction, and (III) after the contraction (Fig. 1b). Both encapsulating droplet and the encapsulated cell change in shape during their passage through the contraction and almost regain their shape downstream the channel. Three different types of leukemia cells in these simulations including, myeloid (HL60) cell line, lymphoid (Jurkat) and neutrophil are modeled as a homogeneous viscoelastic liquid droplet^16,24^. The solvent viscosity of the cell fluid represents the cytosol viscosity and the polymeric viscosity represents the cytoskeleton viscosity^24^. Cells have two viscosities: solvent viscosity representing cytosol viscosity and polymeric viscosity representing cytoskeleton viscosity. Total viscosity is defined as the sum of solvent and polymeric viscosity. Polymeric viscosity ratio is defined as the ratio of polymeric viscosity to the total viscosity. The cell viscoelasticity is modeled by a FENE-CR liquid model^25^, where the rheological characteristics are represented by relaxation time and polymeric viscosity of the fluid. Properties of cells, shell fluid and extracellular fluid are summarized in Table 1. In this model, the cell is initially encapsulated with a shell fluid creating a 36 μm-diameter compound droplet, that flows in FC-40 oil^26^, with the average velocity of $U=0.01$ m/s. FC-40 oil is selected due to its biocompatibility and accessibility^27^. The interfacial tension coefficient of the shell fluid / FC-40 oil interface is set to be $\sigma _o=52$ mN/m^27^. The Reynolds and capillary numbers are calculated based on the extracellular fluid properties ($Re=\rho _oUD/\mu _o=0.37$ and $Ca=\mu _oU/\sigma _o=0.00076$).

**Table 1:** Summary of properties for cells, shell fluid and extracellular fluid.

	Cell type			Encapsulating droplet		Extracellular fluid
	HL60	Neutrophil	Jurkat	Newtonian shell fluid	Viscoelastic shell fluid	FC-40 oil
Density^26,28^ (kg/m^3^)	$1.08\times 10^{3}$	$1.08\times 10^{3}$	$1.08\times 10^{3}$	$10^3$	$10^3$	$1.85\times 10^{3}$
Diameter^29^ (μm)	12.4	8.3	11.5	36	36	−
Cortical tension^29^ (pN/μm)	155	48	21	−	−	−
Polymeric viscosity ratio ^17,30^	0.7	0.7	0.7	−	0.1–0.5	−
Total viscosity ^17,26,30^(*cP*)	40	40	40	2	2	4
Relaxation time^31^ (s)	0.17	0.17	0.17	−	0.1	−

The parameter values are obtained from the references indicated in the table.

### Dynamics of a cell-laden droplet in the constricted microchannel

**Figure 2:**
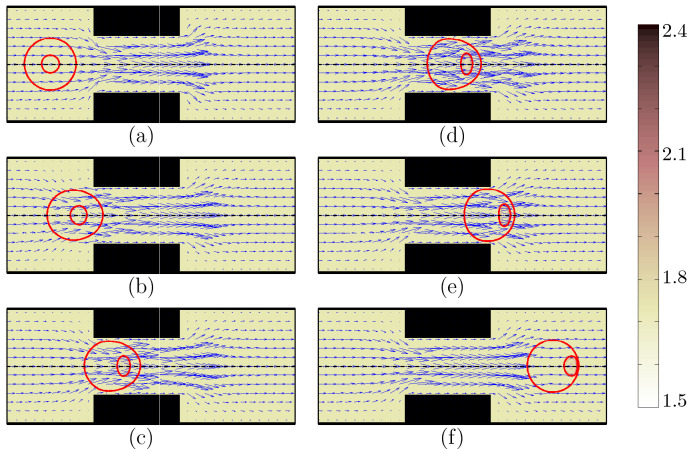
Shape evolution of an encapsulated HL60 through the constricted microchannel at different locations of channel. Velocity vectors are shown in blue to represent the flow dynamics. The red gradient colorbar and contours represent the square root of trace of conformation tensor inside the cell. The viscoelasticity is high around the cell membrane (Ca = 0.00076 and Re = 0.37).

The encapsulated HL60 cell deforms into various shapes as it passes through the constricted channel (Fig. 2a–f) (See Supplementary Video S1. Video is not in real time.). The initially spherical cell-laden droplet (a) moves with the flow, squeezes slowly near the contraction (b) and takes a bullet shape due to the interaction of inertia, interfacial tension and wall forces that results in a complex flow dynamics around the contraction region. The droplet shape becomes asymmetric with a large curvature at the front and a smaller curvature at the rear (c) due to the pressure loss in the flow direction^32^. The encapsulating droplet undergoes a large deformation in the contraction region (d), the deformation is enhanced after the contraction (e) and finally the cell returns to a spheroid shape (f) downstream of the channel.

The flow dynamics inside the compound droplet is also interesting from the point of view of deformation of the encapsulated cell. Inside the encapsulating droplet, the cell moves faster than the droplet. Therefore, the initially concentric compound droplet becomes off-center and the cell gradually reaches the front portion of the droplet, cushioned by a thin layer of liquid. This is due to the pressure loss and the complex flow dynamics inside the droplet^33^. The initial spherical HL60 cell undergoes stresses during its migration in the contraction region which deforms the cell and reshape it to an ellipsoid. Since, the cell moves faster than the encapsulating droplet, it reaches the front of the droplet after passing the contraction and meets the droplet-extracellular fluid interface which enhances the deformation. As the cell moves further downstream, it nearly regains its original shape as a spheroid. The cell experiences viscoelastic stresses through this process which leads to the stretching of long-chain polymers in the cell. The red gradient colorbar and contours in Fig. 2 display the square root of trace of conformation tensor representing the average conformation of coil structure of polymeric particles inside the cells^34^. The cell experiences relatively low viscoelastic stresses which are mostly pronounced around the membrane interface. Velocity vectors are shown in blue to represent the flow dynamics.

### Deformation and viability of different leukemia cell types

**Figure 3:**
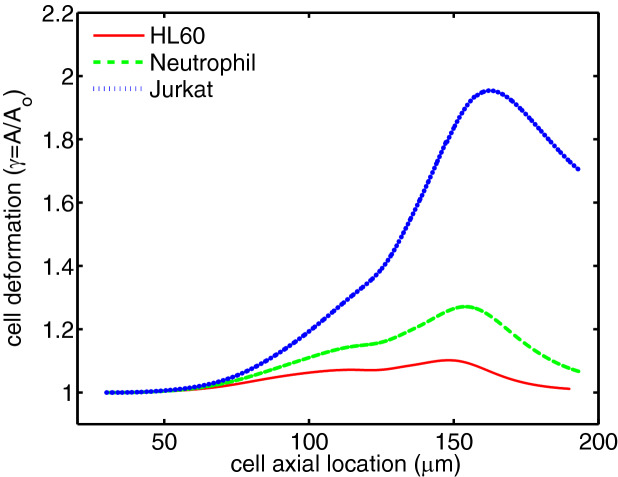
Deformation of different leukemia cells through a constricted microchannel (Ca = 0.00076 and Re = 0.37).

In this section, the motion of the encapsulated HL60, Neutrophil and Jurkat cells in the constricted microchannel and their shape evolution due to mechanical deformation are investigated as they traverse through the channel. These three cell types vary in size and cortical tension (Table 1) that govern the interfacial tension force exerted on the single cell and play important roles in the mechanical deformation of the cell. The cell deformation ($\gamma$) is calculated as the membrane area of the deformed cell (*A*) compared to the membrane area of the undeformed cell ($A_0$), i.e. $\gamma = A/A_0$. This value is equal to unity for an undeformed cell and is always above unity for a deformed cell. As shown in Fig. 3 the three leukemia cells experience similar deformation trends but at different rates, as they pass through the contraction region. Initially, the spherical leukemia cell is encapsulated in the shell fluid and freely moves in the extracellular fluid before entering the contraction region. As this encapsulated droplet enters the contraction region, the cell experiences a small deformation, which grows in size even in the expansion region mainly due to the frontal area of the encapsulating droplet. The maximum deformation for all three cells is observed after the contraction region downstream the channel, where the cell membrane meets shell/extracellular fluid interface. Although the three leukemia cells exhibit similar deformation behavior under stress, their deformation is significantly different in value. The Jurkat cell undergoes maximum deformation and hence is the least viable among the cells. HL60 cell is the least sensitive to deformation, remaining almost alive under high shear stresses around the membrane in the contraction area. This finding emphasizes the importance of cell size and cortical tension in mechanical cell deformation^29^.

Cells are continuously subjected to mechanical stimulation by external forces and when these forces exceed a certain threshold, injury occurs^35^. The mechanical deformation of a cell membrane is a good way to represent mechanical cell injury. We use a theoretical cell survival model to relate cell membrane deformation to cell viability^36^. This theoretical model is based on the experimental data obtained by compressing cells between two parallel plates and it shows that a change in the cell membrane area can result in a rupture and consequently death of the cell^36^. The peak value in cell deformation shown in Fig. 3 indicates the cell viability or the extent to which the cell is damaged or injured. The viability of the three leukemia cells based on the maximum cell deformation $\gamma _{max}$, using this model are: HL60 cells 89.8%; Neutrophil 72.9% and Jurkat 4.6%. According to this model, the Jurkat cell viability of nearly 4.6% threatens its survival as they pass through a narrow contraction. HL60 and Neutrophil appear to survive the narrow part of the channel due to their low deformation.

### Effect of shell fluid viscoelasticity on cell deformation

Viscoelasticity of the shell fluid strongly influences the flow field characteristics inside the encapsulating droplet and affects the shape evolution and the deformation of the encapsulated cell. In particular, high viscoelastic stresses in axial and radial directions due to the high velocity and pressure gradient in the extensional flow around the orifice actively affect the droplet shape in the contraction or expansion part of the microchannels ^37^.

The polymeric concentration of the encapsulating droplet drives the shell fluid rheological behavior. As the polymeric concentration increases, the fluid becomes more viscoelastic with stronger rheological behavior that strongly influences cell-laden droplet dynamics. Polymeric concentration is determined by the amount of polymers suspended in the dilute solution. The polymeric viscosity ratio is defined by $\beta =\mu _{p}/\mu _d$, the ratio of polymeric viscosity to total viscosity, where $\mu _{p}$ and $\mu _{d}$ are polymeric viscosity and total viscosity of the shell fluid, respectively.

**Figure 4:**
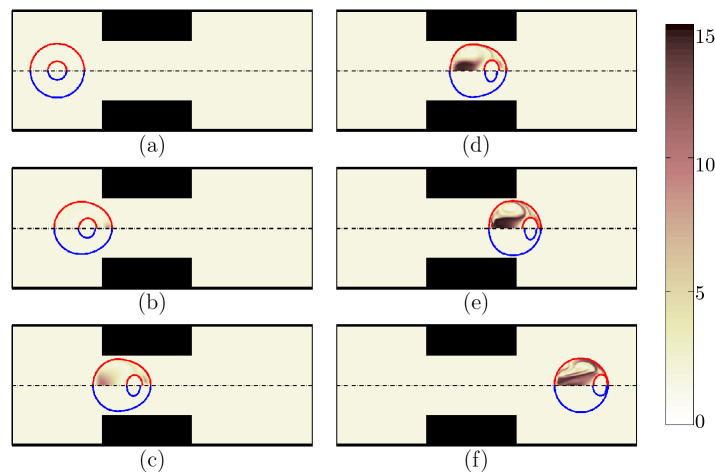
Shape evolution of an encapsulated HL60 through the constricted microchannel at different locations of channel. The shell fluid is Newtonian ($\beta =0$) indicated by blue on the bottom half; the viscoelastic shell fluid ($\beta =0.5$) is indicated by red on the top half. The color bar and contours represent the square root of tensor of conformation tensor inside the cell and the shell fluid ($Ca = 0.00076$ and $Re = 0.37$).

**Figure 5:**
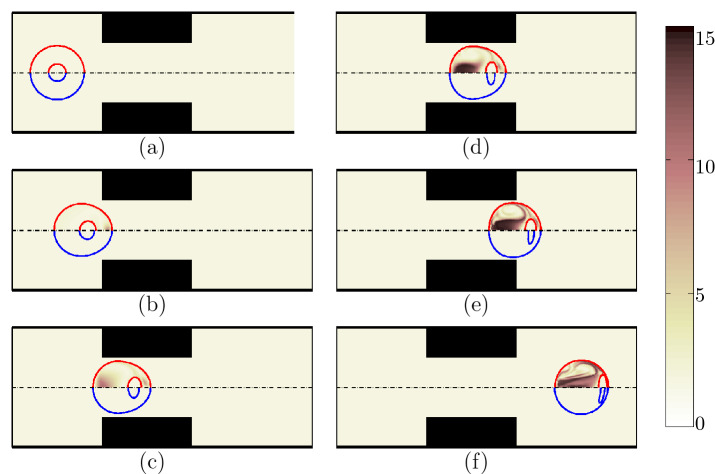
Shape evolution of an encapsulated Jurkat through the constricted microchannel at different locations of channel. The shell fluid is Newtonian ($\beta =0$) indicated by blue on the bottom half; the viscoelastic shell fluid ($\beta =0.5$) is indicated by red on the top half. The color bar and contours represent the square root of trace of conformation tensor inside the cell and the shell fluid ($Ca = 0.00076$ and $Re = 0.37$).

Shape evolution of both encapsulating droplet and the HL60 cell at different locations is shown for Newtonian shell fluid with $\beta =0$ (indicated by the blue circle on the bottom half of Fig. 4) and viscoelastic shell fluid with $\beta =0.5$ (indicated by the red circle on the top half of Fig. 4). The shape evolution of the Jurkat cell is also shown for Newtonian ($\beta =0$) and viscoelastic ($\beta =0.5$) shell fluids in Fig. 5. The relaxation time of the encapsulating droplet is 0.1 s. As the encapsulating viscoelastic shell droplet enters the contraction, viscoelastic stresses are built in the front part of the droplet due to high shear stresses in the constricted area. The stresses are then spread out in the entire encapsulating droplet. These stresses are extremely high around the cell surface and the viscoelastic boundary layer over the cell membrane, absorbs the kinetic energy and protects the cell membrane area against severe mechanical deformation. As a result, the cell encapsulated with a viscoelastic shell fluid deforms less compared to the cell encapsulated with a Newtonian fluid. The color contours in Figs. 4 and 5 show the square root of trace of viscoelastic conformation tensor in the shell fluid and the cell, which represent the average conformation of coil structure of polymeric particles. The high polymer stretching at the rear of the droplet is particularly high due to high axial stresses near the axisymmetric axis, which pulls the interface to a slightly tapered trailing end compared to that for the Newtonian shell fluid ^38^. As the droplet moves into the contraction area, the built-up stresses spread through the entire droplet until after it exits the contraction when the polymer stretching becomes less significant. Comparing the deformation of HL60 (Fig. 4) with Jurkat cells (Fig. 5), the relatively lower cortical tension of the Jurkat cell compared to that of HL60 (Table 1) allows for a high tendency to deform, which results in a strong mechanical injury of the Jurkat cell. Nevertheless, when the shell fluid is viscoelastic surrounding the Jurkat cell, it alleviates the deformation and possibly protect the cell.

**Figure 6:**
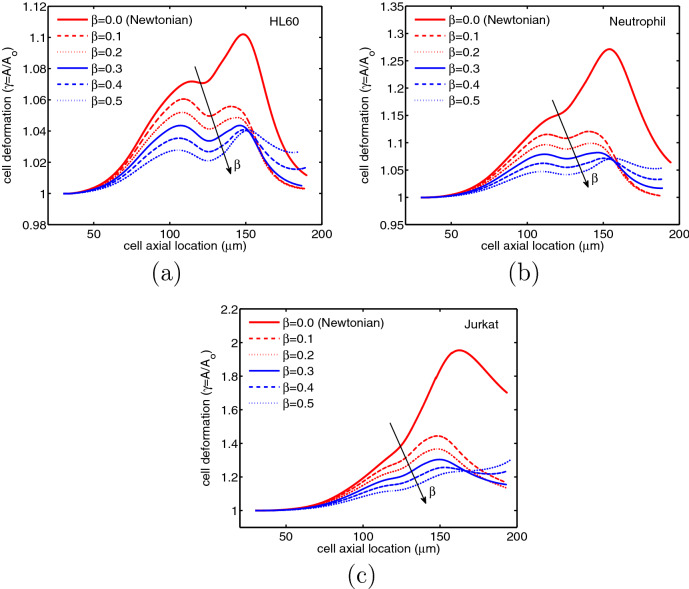
Deformation of (**a**) HL60, (**b**) Neutrophil and (**c**) Jurkat cells at different polymeric concentrations for $Ca=0.00076$ and $Re=0.37$.

Additional simulations are conducted to study the effects of polymeric viscosity ratio on cell deformation. This was done by tuning the polymeric viscosity ratio but by fixing the total viscosity. The cell deformation decreases with increasing polymeric viscosity ratio for HL60, Neutrophil and Jurkat cells (Fig. 6). For all cell encapsulated with either Newtonian or viscoelastic fluids two local extrema in cell deformation can be observed. The first one is associated with the large velocity and stresses in the contraction area and the other local maximum occurs after the cell exits the contraction area, which is associated with the tendency of the cell to move faster than the encapsulating droplet due to pressure loss. Therefore, the cell stays very close to the droplet-extracellular fluid interface and the mechanical deformation increases due to flow constraints. These simulations reveal that the boundary layer between the cell and the viscoelastic droplet creates a separation between the cell and the droplet-extracellular fluid. This results in a smaller second local maximum in cell deformation compared to the Newtonian fluid. The local maximum cell deformation also decreases with increasing polymeric viscosity ratio. From these results, it is possible to infer that the viscoelastic fluids are better barriers to encapsulate cells and reduce cell deformation.

**Figure 7:**
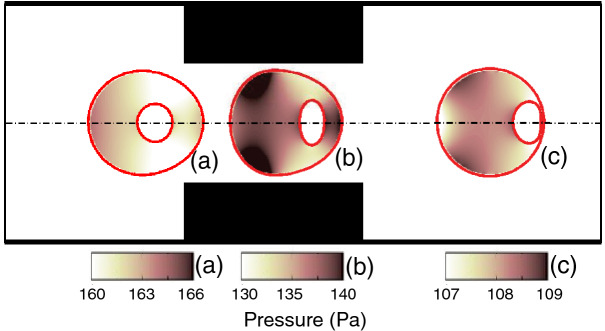
Position of cell and pressure field inside the encapsulating droplet at (**a**) region I: before contraction, (**b**) region II: within the contraction and (**c**) region III: after the contraction. The color bar and contours represent the pressure field inside the encapsulating droplet.

In Fig. 6, a bimodal distribution of cell deformation is observed for all three cell types of encapsulated with Newtonian/viscoelastic fluids. This can be explained using the pressure field in the shell fluid, i.e., in regions I, II and III inside of the encapsulating droplet (Fig. 7). The first of the bimodal peak is due to the sudden contraction in the microchannel at a distance of 60 μm from the entrance, which causes a large cell deformation in the orifice region II. The second peak arises after the droplet passes through the orifice and enters region III around 120 μm from the channel entrance. As the cell-laden droplet travels through the microchannel, the cell position inside the encapsulating droplet changes. As the droplet enters the orifice, the HL60 cell undergoes a slight increase in pressure in the rear of the droplet (Fig. 7a), pushing the position of the cell to the front while deforming the droplet as shown by the first peak in Fig. 6. As the droplet enters the orifice, the pressure is locally high at the rear on either side of the symmetry line in region II, increasing the drag force (Fig. 7b). This pushes the cell further to the front of the droplet, deforming the cell into an oblate spheroid. A similar observation was made by Chaithanya and Thampai^39^ in their analytical study in a compound droplet. Thus, the concentric configuration cannot be maintained, as the cell moves faster than the shell fluid. As the compound droplet moves downstream of the channel into region III, the pressure drop in the shell fluid becomes lower again, however, the shell fluid momentum allows the cell to reach very close to the droplet-extracellular fluid interface (Fig. 7c). As a result, the mechanical deformation increases to form the second peak in the bimodal distribution in Fig. 6.

**Figure 8:**
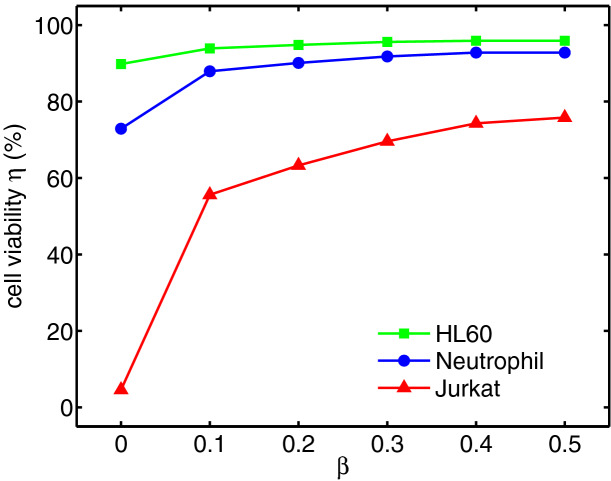
Viability of HL60, Neutrophil and Jurkat cells at different polymeric concentrations for $Ca = 0.00076$ and $Re = 0.37$.

In addition, the results from the viability model shows that by increasing polymer concentration, the cell deformation can be controlled to protect cells from severe damage or extinction (Fig. 8). The HL60 and Neutrophil cells remain alive, with only a slight change in viability with increasing polymeric viscosity ratio. However, the Jurkat cell is highly affected when it is surrounded by Newtonian shell fluid, and the viability increases with increasing polymeric viscosity ratio as is the case with all cells (See Supplementary Videos S1, S2, S3. Videos are not in real time.).

## Discussion

Flow dynamics, deformability and viability of encapsulated leukemia cells migrating through a constricted microcapillary is simulated numerically. All three cells studied here, myeloid (HL60) cell line, lymphoid (Jurkat) and neutrophil tend to elongate due to the stresses and forces exerted on their membrane area. Initially spherical encapsulated cells experience high pressure and velocity gradients when they migrate in a constricted channel. These high gradients along with the wall effects of the channel deform the cell. After a certain threshold, the mechanical deformation causes cell rupture and decreases the cell viability. The three leukemia cells elongate while passing through the microchannel constriction and again regain their shape downstream of the channel. However, their deformation characteristics are different, with the Jurkat cells being the least viable due to dramatically high deformation at low polymer concentration. This is mainly essentially due to lower cortical tension of the Jurkat cell compared to that of the other two cells.

Rheology of the encapsulating droplet is found to protect the cells against massive mechanical deformation. The shell fluid viscoelasticity can be tuned by changing the polymeric viscosity ratio. The viscoelasticity and elastic recoil of polymer solutions due to high velocity and shear stresses in the shell fluid protect the cell and assure its survival. It is found that for all three leukemia cells, increasing the polymeric viscosity ratio results in smaller deformation of the cells regardless of their size and other properties such as cortical tension. In summary, the viability of cells can be tuned by engineering the rheology of shell fluid. Increasing the shell fluid polymeric concentration minimizes mechanical cell deformation and as a result improves cell viability.

## Methods

### Viscoelastic liquid droplet model for leukemia cells

The cells and biocompatible fluids have been modeled as viscoelastic fluids. In these models, the cell, which resembles a viscoelastic liquid droplet, has a Newtonian component (cytosol) and a viscoelastic component (cytoskeleton)^16,24,31^. The liquid droplet model with a constant cortical tension has been previously used to model the cell ^16,29,40^. However, this model for leukemia cells rests on two main assumptions: the internal contents of the cell are homogenous liquid, and the cortical tension is constant around the cell^29^. Cells have polymeric components such as cytoskeleton which characterize their rheological behavior and need to be accounted for in order to be accurately modeled. Therefore, we model the cell as a viscoelastic liquid droplet. In this model the cell viscosity has two components: solvent viscosity which represents cytosol viscosity and polymeric viscosity which represents cytoskeleton^24^. The viscoelasticity inside the cell is represented by a FENE-CR viscoelastic model (Finite Extendable Nonlinear Elastic-Chilcott and Rallison)^25^. The FENE-CR model is chosen here because it can represent relaxation mechanism and finite extensibility of protein-based components of the cell. In a FENE-CR liquid, polymers are modeled as finitely extensible spring dumbbells in a solution, which is deemed to be more realistic than the Maxwell and Oldroyd-B models where the polymers are assumed to be infinitely extensible^41^.

### Numerical method

The incompressible Navier–Stokes equations which govern the fluid flow in the microchannel are solved in the framework of a one-field formulation on an Eulerian grid for all three phases including the cell, the shell fluid and the extracellular fluid. These continuity and momentum equations can be expressed as:

1 $$\begin{aligned} \nabla \cdot \mathbf{u }= & {} 0, \end{aligned}$$

2 $$\begin{aligned} \frac{\partial \rho \mathbf{u} }{\partial t} + \nabla \cdot (\rho \mathbf{uu })= & {} -\nabla p + \nabla \cdot \mu _{s}(\nabla \mathbf{u} +\nabla \mathbf{u }^T) + \nabla \cdot \pmb {\tau } + \int _A \sigma \kappa {\mathbf{n}}\delta ({\mathbf{x}} - {\mathbf{x}}_{\mathbf{f}})dA, \end{aligned}$$

where $\rho$, $\mu _s$, *p* and **u** denote the density, solvent viscosity, pressure, velocity vector, and $\pmb {\tau }$ is the viscoelastic extra stress tensor. The last term in the momentum equations represents the interfacial tension, where $\sigma$ is the interfacial tension coefficient, $\kappa$ is the mean curvature, $\mathbf{n}$ is the outward unit vector normal to the interface, and $\delta$ is the three-dimensional delta function. The interfacial tension force acts only on the interface location denoted by $\mathbf{x}_f$ which is solved on a Lagrangian grid and then is projected on the Eulerian grid to discrete momentum equation. Both the cell and encapsulating fluids are modeled as viscoelastic liquids using the FENE-CR model given by:

3 $$\begin{aligned} \frac{\partial \mathbf{A }}{\partial t} + \nabla \cdot (\mathbf{u }\mathbf{A }) - (\nabla \mathbf{u })^T\cdot \mathbf{A } - \mathbf{A }\cdot \nabla \mathbf{u }= & {} - \frac{L^2}{L^2-{\text{trace}}(\mathbf{A })} \frac{\mathbf{A } - \mathbf{I }}{\lambda }, \end{aligned}$$

4 $$\begin{aligned} \pmb {\tau }= & {} \mu _{p} \left( \frac{L^2}{L^2-{\text{trace}}(\mathbf{A })}\right) \frac{\mathbf{A } - \mathbf{I }}{\lambda }, \end{aligned}$$

where **A**, $\lambda$, *L*, **I**, and $\mu _p$ are the conformation tensor, the relaxation time, the extensibility parameter (i.e., the ratio of the length of a fully extended polymer dumbbell to its equilibrium length), the identity tensor, and polymeric viscosity, respectively. Following Izbassarov and Muradoglu^45^, the extensibility parameter for the cell and the encapsulating droplet fluid is assumed to be the same and specified as $L=15$. For a Newtonian fluid, polymeric viscosity is zero. Therefore, the viscoelastic stress tensor in FENE-CR model, calculated by Eq. (4), becomes zero and as a result the viscoelastic term ($\nabla$.$\pmb {\tau }$) in the Navier–Stokes equation vanishes. The conformation tensor (**A**) which represents the deformation of polymer molecules in the flow is calculated within the cell and encapsulating shell fluid (Eq. (3)), which will be then incorporated to calculate viscoelastic extra stress tensor ($\pmb {\tau }$) (Eq. (4)). Note that the conformation tensor (**A**) is a positive definite tensor representing the average conformation of coil structure of polymers during flow. The eigenvalues of the conformation tensor and their corresponding eigenvectors have the physical meaning of square of the average macromolecular size along the principle three directions and the orientation of those directions in space, respectively. All those eigenvalues need to be positive, i.e. the conformation tensor needs to be positive definite^42,43^.

The Navier–Stokes equations (1) and (2) are solved in the entire computational domain. Note that the flow is assumed to be axisymmetric, therefore only one half of the microchannel is used as the computational domain. At the inlet of the microchannel, the flow is initiated by imposing fully developed velocity profile, with an average velocity of 0.01 m/s. The pressure is constant at the outlet, and symmetry and no-slip boundary conditions are used at the centerline and on the wall of microchannel, respectively. The multiphase flow is resolved by a viscoelastic front-tracking method^44^ that was developed by Izbassarov and Muradoglu^45^. In this method the field quantities are solved on a staggered Eulerian grid and the interfaces between different phases are tracked by a Lagrangian grid consisting marker points connected with elements which move by the calculated velocity field on the Eulerian grid^44–46^. The spatial and time derivatives are approximated using central differences and first order explicit method, respectively. The FENE-CR model Eqs. (3) and (4) are solved within the cell and viscoelastic shell fluid droplet, where the viscoelasticity appears. The viscoelastic convective term in viscoelastic model is treated by a fifth-order upwind WENO-Z scheme along with a log-conformation method to overcome high Weissenberg number to preserve the positive-definiteness of the conformation tensor^45,46^.

### Cell viability model

The mechanical deformation of the cell is quantified by the relative cell membrane area (i.e. $\gamma = A/A_0$), where *A* and $A_0$ are the surface areas of the deformed and the undeformed cells, respectively. Then a cell viability model proposed by Takamatsu and Rubinsky^36^ is exploited to compute the cell viability and to identify the extent of cell damage due to mechanical deformation. This theoretical model is derived based on experimental data of cell deformation during compression between two plates which essentially measured the percentage of impaired cells to total cells stained by trypan blue. A change in cell surface area results in rupture and consequently reduces the cell viability. This cell viability can be formulated based on the maximum instantaneous cell deformation ($\gamma _{max}$), as:

5 $$\begin{aligned} \eta (\gamma ) = \left\{ \begin{array}{ll} 1&{}\text {for}\;\; \gamma _{\text{max}} <\gamma _{cr}-\Delta \gamma ,\\ \frac{1}{2}-\frac{\gamma _{\text{max}} -\gamma _{cr}}{2\Delta \gamma }&{}\text {for}\;\; \gamma _{cr}-\Delta \gamma \le \gamma _{\text{max}} \le \gamma _{cr}+\Delta \gamma ,\\ 0&{} \text {for} \;\;\gamma _{\text{max}}>\gamma _{cr}+\Delta \gamma , \end{array} \right. \end{aligned}$$

With $\gamma _{cr} = 1.5$ and $\Delta \gamma = 0.5$ as the critical cell deformation and the range of surface expansion, respectively^17, 36^.

## Supplementary information

Supplementary Video S1

Supplementary Video S2

Supplementary Video S3
